# Systematic Proteome Profiling of Maternal Plasma for Development of Preeclampsia Biomarkers

**DOI:** 10.1016/j.mcpro.2024.100826

**Published:** 2024-08-05

**Authors:** Ji Hyae Lim, Jae Min Lim, Hyeong Min Lee, Hyun Jung Lee, Dong Wook Kwak, You Jung Han, Moon Young Kim, Sang Hee Jung, Young Ran Kim, Hyun Mee Ryu, Kwang Pyo Kim

**Affiliations:** 1 Smart MEC Healthcare R&D Center, CHA Future Medicine Research Institute, CHA Bundang Medical Center, Seongnam, Republic of Korea; 2 Department of Applied Chemistry, Institute of Natural Science, Global Center for Pharmaceutical Ingredient Materials, Kyung Hee University, Yongin, Republic of Korea; 3 Department of Obstetrics & Gynecology, CHA Ilsan Medical Center, CHA University, Gyeonggi-do, Republic of Korea; 4 Department of Obstetrics and Gynecology, Ajou University School of Medicine, Gyeonggi-do, Republic of Korea; 5 Department of Obstetrics and Gynecology, CHA Gangnam Medical Center, CHA University School of Medicine, Seoul, Republic of Korea; 6 Department of Obstetrics and Gynecology, CHA Bundang Medical Center, CHA University School of Medicine, Seoul, Republic of Korea; 7 Department of Biomedical Science and Technology, Kyung Hee Medical Science Research Institute, Kyung Hee University, Seoul, Republic of Korea

**Keywords:** preeclampsia, proteomics, biomarkers, plasma, LC-MS/MS

## Abstract

Preeclampsia (PE) is a hypertensive disorder of pregnancy with various clinical symptoms. However, traditional markers for the disease including high blood pressure and proteinuria are poor indicators of the related adverse outcomes. Here, we performed systematic proteome profiling of plasma samples obtained from pregnant women with PE to identify clinically effective diagnostic biomarkers. Proteome profiling was performed using TMT-based liquid chromatography-mass spectrometry (LC-MS/MS) followed by subsequent verification by multiple reaction monitoring (MRM) analysis on normal and PE maternal plasma samples. Functional annotations of differentially expressed proteins (DEPs) in PE were predicted using bioinformatic tools. The diagnostic accuracies of the biomarkers for PE were estimated according to the area under the receiver–operating characteristics curve (AUC). A total of 1307 proteins were identified, and 870 proteins of them were quantified from plasma samples. Significant differences were evident in 138 DEPs, including 71 upregulated DEPs and 67 downregulated DEPs in the PE group, compared with those in the control group. Upregulated proteins were significantly associated with biological processes including platelet degranulation, proteolysis, lipoprotein metabolism, and cholesterol efflux. Biological processes including blood coagulation and acute-phase response were enriched for down-regulated proteins. Of these, 40 proteins were subsequently validated in an independent cohort of 26 PE patients and 29 healthy controls. APOM, LCN2, and QSOX1 showed high diagnostic accuracies for PE detection (AUC >0.9 and *p* < 0.001, for all) as validated by MRM and ELISA. Our data demonstrate that three plasma biomarkers, identified by systematic proteomic profiling, present a possibility for the assessment of PE, independent of the clinical characteristics of pregnant women.

## Highlights

• Global proteome analysis quantified 138 proteins in preeclampsia.
• APOM, LCN2, QSOX1 validated via MRM showed high PE detection accuracy.
• ELISA validation of APOM, LCN2, QSOX1 confirmed high diagnostic accuracy.

## Graphical Abstract

**Figure:**
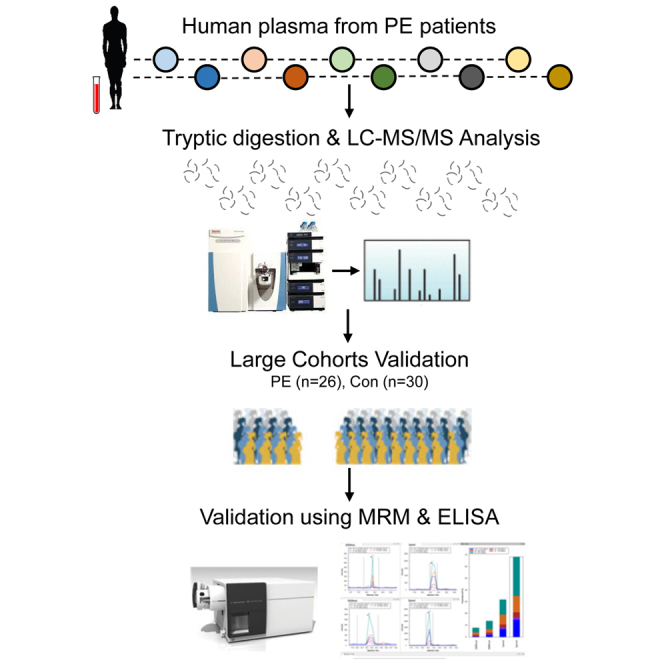

## In Brief

Traditional biomarkers for preeclampsia (PE) overlap with other diseases like high blood pressure and proteinuria. Here, after proteome profiling using TMT-based liquid chromatography-mass spectrometry (LC-MS/MS) followed by validation using multiple reaction monitoring (MRM) analysis and enzyme-linked immunosorbent assay (ELISA), it has been identified that three markers, APOM, LCN2, and QSOX1, showed high diagnostic accuracies for PE detection.

Preeclampsia (PE) is a hypertensive pregnancy disorder affecting approximately 2 to 8% of all pregnancies globally and is one of the leading causes of maternal and neonatal mortality and morbidity (1). It is a multisystem disorder characterized by the onset of hypertension and proteinuria after 20 weeks of gestation. However, the poor diagnostic capability of high blood pressure and proteinuria, which are traditional markers for PE, is related to adverse outcomes of PE. According to the recently updated guideline of the American College of Obstetricians and Gynecologists, even in the absence of proteinuria, the finding of new-onset hypertension with maternal organ dysfunction including thrombocytopenia, renal insufficiency, impaired liver function, and pulmonary edema is diagnosed to PE. These indicators are recommended for sub-classifying the severity of PE (1). In the past decade, various biomarkers have been developed and studied in an attempt to ameliorate the diagnosis and management of PE. Angiogenic biomarkers, such as soluble FMS-like tyrosine kinase and placental growth factor, in the serum, are recognized as clinically sensitive and specific biomarkers of PE (2, 3, 4). Nevertheless, the search for superior biomarkers for PE detection is ongoing and new studies are constantly being published (5, 6, 7).

The use of biofluid (*e.g.*, serum or urine) for the analysis of naturally occurring proteome as a source of biomarkers has been reported for various diseases (8, 9, 10, 11). For clinical application, mass spectrometry (MS)--based profiling of naturally occurring proteins can provide an extensive inventory of serum peptides derived from either highly abundant endogenous circulating proteins or cell and tissue proteins. These peptides are usually soluble and stable in the presence of endogenous proteases or peptidases, thus, they can be directly used for liquid chromatography-mass spectrometry (LC-MS) analysis without additional manipulation, such as tryptic digestion. In a previous study to identify diagnostic biomarkers for PE, 612 serum peptides were analyzed by MS (12). The study demonstrated that 19 peptides derived from six proteins were accurate indicators of PE. Recently, a plasma peptides-based proteome profiling approach was attempted for the discovery of PE biomarkers (12). However, multiplex proteomic panels using MS can present analytical validation challenges in the clinical laboratory. Therefore, systematic proteome profiling such as multiple reaction monitoring (MRM) analysis using high-resolution LC-MS/MS may improve our understanding of the development of the biomarkers to effectively diagnose PE in pregnant women.

Here, we performed global proteomic profiling of plasma from pregnant women with and without PE, using LC-MS/MS and MRM assay, to identify plasma biomarkers with differential proteomic signatures. The plasma diagnostic biomarkers for PE identified in the study were subsequently validated in an independent patient group using enzyme-linked immunosorbent assay (ELISA).

## Experimental Procedures

### Experimental Design and Statistical Rationale

For sequential proteomic profiling analysis using LC-MS/MS, plasma samples from participants with preeclampsia (PE, n = 4) and severe-preeclampsia (SPE, n = 7) according to their severity and control group (n = 3) were randomly selected from all patient plasma samples and used for biomarker discovery. All plasma samples were digested into peptides and labeled with Tandem Mass Tags (TMTs). A total of 4 sets of TMT 6plex were used and the global standard was included to each set to normalize the variation between TMT sets. All samples were digested and labeled with tandem mass tag (TMT) and pooled. The pooled sample was then divided into 12 fractions by high pH fractionation. All the fractions were subsequently injected into the LC-MS/MS system, and the raw mass spectra were processed by Proteome Discoverer v2.1. The statistical significance of differential peptide expression has been measured using Student’s *t* test. MRM-based LC-MS/MS in an independent cohort of PE (n = 26) and control (n = 29). Heavily labeled standards of each target peptide were mixed and spiked into each cohort. Samples were digested and subsequently injected into the LC-MS/MS system, and the raw mass spectra were processed by Skyline 23.1. Peptide concentration was calculated using light and heavy abundance ratios. The clinical accuracy of the novel-identified biomarkers was further validated in an independent patient group comprising pregnant women with PE (n = 10) and controls (n = 30).

### Study Subjects and Sample Processing

This study was approved by the Institutional Review Board (IRB) and the Ethics Committee of Cheil General Hospital (#CGH-IRB-2017-22) and carried out according to the Declaration of Helsinki protocols. Singleton pregnant women who attended antenatal care at the Cheil General Hospital’s Department of Obstetrics and Gynecology were enrolled in this study. Written informed consent was obtained from all participants before the collection of samples and subsequent analysis.

A total of 109 maternal plasmas from 62 pregnant women without PE (Controls) and 47 pregnant women with PE were used. Based on the basic clinical characteristics of PE, PE patients were classified into hypertension and proteinuria. The definition of study subjects was described previously (13). In brief, PE was defined as hypertension [systolic blood pressure (SBP) ≥ 140 mmHg and/or diastolic blood pressure (DBP) ≥ 90 mmHg on at least two occasions 4 h apart] and proteinuria (≥300 mg in a 24 h urine collection specimen and/or ≥ 1+ on dipstick testing) after 20 weeks of gestation. PE was subcategorized as “PE” and “PE with severe features (SPE)”. The severe features of PE were defined as DBP ≥110 mmHg, SBP ≥160 mmHg, onset before 34 weeks of gestation, or a birth weight below the 10th percentile based on gender and gestational age at birth. Control cases were defined as women without medical and obstetric complications who presented for delivery at term (≥37 weeks of gestation). The cases of pregnancies with multiple gestations, aneuploidy, major fetal abnormalities, miscarriage before 20 weeks of gestation, stillbirth after 20 weeks of gestation, chronic hypertension, prior history of PE, gestational hypertension without proteinuria, renal disease, pregestational diabetes mellitus, or gestational diabetes mellitus were excluded from this study.

Maternal peripheral blood was obtained by venipuncture and collected in tubes containing EDTA when hospitalized for delivery. The samples were centrifuged at 1600*g* for 10 min, and the supernatant was then re-centrifuged at 16,000*g* for 10 min and aliquoted for proteomic analysis and validation of biomarkers. These samples were stored at – 80 °C until required.

### Removal of High Abundance Proteins

Removal of high-abundance proteins (for example, albumin, IgG, transferrin, and complement C3) from plasma samples was conducted using the Seppro IgY14 removal column (Sigma Aldrich, SEP020). In the Agilent Infinity 1290 high-performance liquid chromatography (HPLC), the IgY 14 removal column was linked and the plasma protein sample was injected. Seppro dilution buffer, stripping buffer, and neutralization buffer were used, and a linear gradient was set as follows: 0 to 17 min, dilution buffer (100%), 0.2 ml/min; 17.01 to 22 min, dilution buffer (100%), 1.5 ml/min; 22.01 to 36 min, stripping buffer (100%), 1.5 ml/min; 36.01 to 42 min, neutralization buffer (100%), 1.5 ml/min; 42.01 to 50 min, dilution buffer (100%), 1.5 ml/min. Two major peaks were observed; the fractions corresponding to the first peak were collected as low-abundance proteins, and those corresponding to the second peak were abandoned as high-abundance proteins.

### Protein Digestion

The collected low-abundance proteins were concentrated using a bicinchoninic acid (BCA) assay, and 100 μg protein of each sample was denatured using 8 M urea with sonication for 30 min, and then reduced by incubation with 1 M dithiothreitol at 37 °C for 45 min. The reduced proteins were alkylated with 1M iodoacetamide in the dark for 30 min and then digested by incubation with trypsin (ThermoFisher Scientific) at a ratio of 50:1 at 37 °C overnight. The digested peptides were desalted using a Harvard spin column cartridge according to the manufacturer’s instructions, dried, and stored at –80 °C.

### Tandem Mass Tag (TMT) Labeling and High-pH Fractionation

Dried peptides were resolved in 100 μl of 100 mM triethylammonium bicarbonate (TEAB) and a BCA assay was performed to determine the reconstituted peptide concentration. A pooled sample for normalization between TMT sets was prepared by combining tryptic peptides from each individual sample. The peptide samples were labeled with the TMTsixplex isobaric label reagent set (ThermoFisher Scientific), from 126 to 128 for PE, 130 for the control, and 131 for the global reference. TMT reagents were solved in 80 μl of acetonitrile (ACN), 40 μl was added to each sample, followed by incubation at room temperature for 1h. Labeled peptides were pooled together for high-pH fractionation.

The pooled sample was fractionated using a Thermo UltiMate 3000 HPLC system (ThermoFisher Scientific), based on the fractionation method of basic pH reverse-phase liquid chromatography. The Xbridge C18 column (4.6 mm × 250 mm, 130 A, 5 μm) was used and Solvents A and B were 10 mM ammonium formate in water (pH 10) and 10 mM ammonium formate in 90% ACN (pH 10), respectively. The total run time was 110 min and the gradient was set as follows (T min/% of solvent B): 10/0, 20/10, 80/35, 95/70, 105/70, and 115/0. In total, 96 fractions were collected every minute between 14 min and 110 min and non-contiguously pooled into 12 fractions. Fractionated peptides were desalted, dried, and stored at – 80 °C.

### LC-MS/MS Profiling Analysis

The fractionated peptides were analyzed using a Q Exactive Orbitrap mass spectrometer coupled with a nanoACQUITY UPLC (Waters) as our previous study (14). For the proteome profiling analysis, the UPLC gradient was set as follows (T min/% of solvent B): 0/5, 5/10, 100/40, 102/80, 112/80, 114/5, and 120/5. The peptides were ionized through an EASY-spray column (50 cm × 75 μm ID) packed with 2 μm C18 particles at an electric potential of 1.8 kV. The full MS scan range was set to 400 to 2000, and the resolution was set to 70,000 at m/z 200. The automated gain control target value was 1.0 × 10^6^ during a maximum ion injection time of 100 ms, and Normalized Collision Energy (NCE) was set to 30%. Dynamic exclusion time was set to 30 s. For MS/MS, the resolution was 17,500 and the maximal ion injection time was 50 ms.

### Raw Data Processing and Data Analysis

The raw data acquired from LC-MS/MS analysis were analyzed using Proteome Discoverer (version 2.1), with the Sequest HT search engine. The human database was acquired from UniProt (Homo Sapiens, UP000005640) database containing 20,158 genes. proteins were identified through the peptide sequences matched from the LC-MS/MS spectrum. Search conditions were limited to peptides that have a length of 6 to 144 and missed cleavage was allowed up to two. MS1 tolerance was limited to 10 ppm and MS2 to 0.02 Da. Methionine Oxidation was considered as variable modification, cysteine alkylation, and N-termini, lysine TMT 6plex were considered as static modification. False discovery rate (FDR) is applied at 1% each at the spectrum, peptide, and protein levels.

### MRM Analysis

The digested peptides were analyzed using an Agilent Triple Quadrupole 6490 mass spectrometer coupled with Agilent 1290 Infinity HPLC and using a dynamic multiple reaction monitoring (dMRM) method. The input to the dMRM consisted of a total of 123 transitions from 41 peptides, with a dwell time set to 20 ms for each transition. According to HPLC conditions, the gradient was set as follows (T min/% of solvent B): 0/5, 5/10, 45/35, 46/80, 50/80, 51/5, and 60/5. Peptides were eluted through an ACQUITY UPLC peptide BEH C18 column (1.7 μm, 2.1 mm × 250 mm) with the sheath gas temperature set as 100 °C. Heavily labeled standards of each target peptide were mixed and spiked into each cohort to calculate the concentration of each peptide, and the raw mass spectra were processed by Skyline 23.1.

### Validation of PE Biomarker Candidates Using ELISA

The identified biomarkers for the assessment of PE were validated using commercial ELISA kits as per the manufacturer’s instructions. All assays were performed to measure the plasma levels of the candidate biomarkers in the PE and control subjects of the independent cohort. The receiver–operating characteristics (ROC) curve analysis was performed to assess the optimal cutoff value of the candidate biomarkers. The optimal cutoff was set at 90% sensitivity for each biomarker. The specificity was calculated to consider the diagnostic efficiency using MedCalc (https://www.medcalc.org/calc/diagnostic_test.php) and compared at an equivalent sensitivity. The overall accuracy of the biomarkers was estimated with the area under the curve (AUC) for ROC with a 95% confidence interval (CI). Values of *p* < 0.05 were considered statistically significant.

### Equation to Detect PE Using a Combination of Novel Biomarkers

In this study, we used the concentration of each biomarker to construct the detection equation, which can be used to classify cases. We hypothesized that a combination of novel biomarkers may be able to achieve accurate detection of PE with confidence. To test this, we used statistical tools to generate the ideal combination of novel biomarkers and then calculated the discriminant function coefficients for each of the biomarkers. Multiple regression analysis was used to construct optimal fitting models for multiple independent variables, as described in our previous study (15). The discriminating value (D value) resulting from the detection equation was then measured. We classified the cases that produced a D value above the cutoff value as PE, and cases with values below the cutoff value as normal. The ROC curve analysis was performed to assess the optimal cutoff value of the D value.

### Statistical Analysis

Descriptive data were presented as the means with standard deviation and categorical variables as proportions and counts. Fisher’s exact test was used for the comparison of categorical variables across the clusters, and Student’s *t* test was used for continuous variables. Statistical analyses were performed with the Statistical Package for the Social Sciences version 25.0 (SPSS Inc.).

## Results

### Clinical Characteristics of the Study Groups

The clinical characteristics of the study groups are summarized in Table 1 and Supplemental Table S1. Maternal age, gravidity, and the percentage of nulliparity were not different between the control and PE groups (*p* > 0.05 for all). However, prepregnancy body mass index (BMI) was higher in the PE group than in the control (*p* < 0.001). Blood pressure measured at the highest blood pressure in pregnancy was higher in the PE group than in the control group (*p* < 0.001). Proteinuria was detected in the PE group only. In terms of pregnancy outcome, the gestational age at delivery, the preterm delivery rate, the birth weight of babies, and the percentage of NICU admission were significantly different in the PE, to those in the controls (*p* < 0.001 for all). However, there was no significant difference in the gender ratio of the babies between the study groups (*p* = 0.250).

**Table 1:** Patient demographics and characteristics

Characteristics	Con (n = 62)	PE (n = 47)	*p* value^a^
Maternal age, years	34.8 ± 3.9	35.3 ± 4.0	0.617
Prepregnancy body mass index, kg/m^2^	21.2 ± 2.9	23.9 ± 3.9	<0.001
Prepregnancy maternal weight, kg	55.5 ± 7.1	62.8 ± 9.5	<0.001
Gravidity, n	2.0 ± 0.9	1.8 ± 1.4	0.363
Nulliparity, % (n)	56.5 (35)	70.2 (33)	0.165
Blood pressure and Proteinuria			
Highest SBP in pregnancy, mmHg	118.3 ± 10.2	153.2 ± 17.0	<0.001
Highest DBP in pregnancy, mmHg	72.2 ± 8.5	96.8 ± 11.0	<0.001
Proteinuria at PE presentation (dipstick result: range)	0	2.4 ± 1.1	<0.001
Pregnancy outcome			
Gestational age at delivery, weeks	39.0 ± 1.1	37.2 ± 1.7	<0.001
Delivery <37 weeks, % (n)	0 (0)	27.7 (13)	<0.001
Birth weight of baby, kg	3.4 ± 0.4	2.6 ± 0.7	<0.001
NICU admission, % (n)	3.2 (2)	34.0 (16)	<0.001
Male infant, % (n)	56.4 (35)	44.7 (21)	0.250

Continuous variables are presented as the mean ± standard deviation and discrete variables are presented as percentages (n).

SBP: systolic blood pressures, DBP: diastolic blood pressures, NICU: neonatal intensive care unit.

a Calculated using Fisher’s exact tests for categorical variables and a Student’s *t* test for continuous variables.

### Comprehensive Plasma Proteomic Profiling

**Fig. 1:**
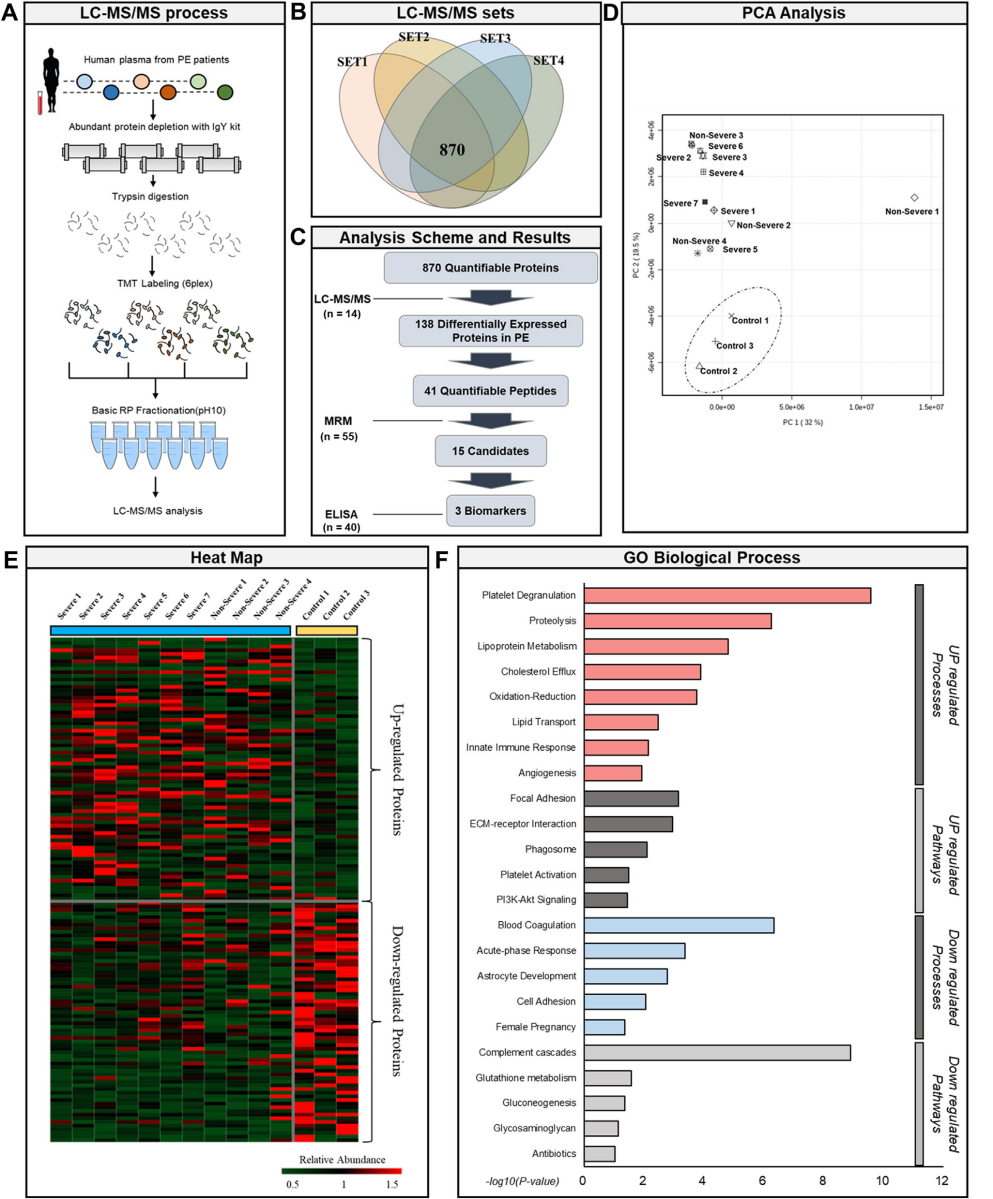
**A Study schema for plasma proteome profiling**. *A*, liquid chromatography-mass spectrometry (LC-MS/MS) process, (*B*) LC-MS/MS analysis sets: Set 1 (PE 1–3), Set 2 (Control 1, PE 4–6), Set 3 (Control 2, PE 7–9), Set 4 (Control 3, PE 10–11). *C*, analysis scheme summary. *D*, Principle Component Analysis (PCA) of plasma profiling data. *E*, heat map of differentially expressed proteins. *F*, Gene Ontology (GO) analysis of differentially expressed proteins (DEPs) in preeclampsia (PE) (GRCh37/hg19 assembly).

Initially, to identify the proteins showing significant differences between the PE and control samples, we performed TMT-based LC-MS/MS. A total of 14 plasma samples, comprising eleven samples (four PE and seven SPE) from PE patients with different clinical characteristics according to PE severity and three from healthy donors, and one pooled sample for normalization between TMT sets were profiled to select DEPs from an in-depth plasma proteome in PE (Fig. 1*A*). The analysis was performed with a total of four TMT 6-plex experiments: Set 1 (PE 1 & 2, SPE 1), Set 2 (Control 1, SPE 2–4), Set 3 (Control 2, PE 3, SPE 5 & 6), Set 4 (Control 3, PE 4, SPE 7) (Fig. 1*B*). We identified a total of 1307 proteins (FDR <0.01) across all samples in the four TMT 6-plex experiments by LC-MS/MS followed by quantification of 870 common proteins (Fig. 1, *B* and *C*). Through the Principal Component Analysis (PCA), we confirmed that the control and PE group were completely distinguished, showing 51.5% of the variance in PC1 and PC2 (Fig. 1*D*). However, there was no significant difference in plasma protein expression in PE and SPE groups. As a result, PE and SPE were grouped together as a PE group and used for further analysis.

Log_2_-transformed relative protein expression was indicated in the heatmap. The blue color indicates PE (severe and non-severe), and the yellow color indicates the control group. Pearson correlation was used for clustering of proteins, distinguishing PE and control groups (Fig. 1*E*). The differentially expressed proteins (DEPs) in PE were selected based on the fold changes ( ≥ 1.3, ≤ 0.7) and *p*-value (<0.05) cut-off value. A total of 138 DEPs were identified of which 71 DEPs (51%) were upregulated and 67 DEPs (49%) were downregulated in PE, compared to the controls (Supplemental Tables S2 and S3). In addition to the identification of proteins reported to be differentially expressed plasma proteins in PE, such as PAPPA and FLT1, novel proteins were identified including A2M, PRG2, and HEXB. Further analysis of the biological functions of DEPs revealed that the upregulated DEPs in PE were related to biological processes, such as platelet degranulation (n = 10, *p* = 2.7E-10), proteolysis (n = 13, *p* = 5.8E-7), lipoprotein metabolism (n = 5, *p* = 1.5E-5), cholesterol efflux (n = 4, *p* = 1.3E-4), oxidation-reduction (n = 5, *p* = 1.7E-4), lipid transport (n = 4, *p* = 3.5E-3), innate immune response (n = 7, *p* = 7.3E-3), and angiogenesis (n = 5, *p* = 1.2E-2), whereas the downregulated DEPs were associated with blood coagulation (n = 9, *p* = 4.6E-7), acute-phase response(n = 3, *p* = 4.3E-4), astrocyte development (n = 3, *p* = 1.7E-3), cell adhesion (n = 3, *p* = 8.7E-3), and female pregnancy (n = 3, *p* = 4.5E-2) (Fig. 1*F*). Moreover, upregulated DEPs in PE were related to the biological pathways such as focal adhesion (n = 7, *p* = 7.1E-4), ECM-receptor interaction (n = 5, *p* = 1.1E-3), phagosome (n = 5, *p* = 8.2E-3), platelet activation (n = 4, *p* = 3.2E-2), and PI3K-Akt signaling (n = 6, *p* = 3.5E-2). Downregulated DEPs were also related to the complement cascades (n = 9, *p* = 1.3E-9), glutathione metabolism (n = 3, *p* = 2.7E-2), gluconeogenesis (n = 3, *p* = 4.5E-2), glycosaminoglycan biosynthesis (n = 2, *p* = 7.4E-2), and antibiotics (n = 4, *p* = 9.2E-2) (Fig. 1*F*).

**Fig. 2:**
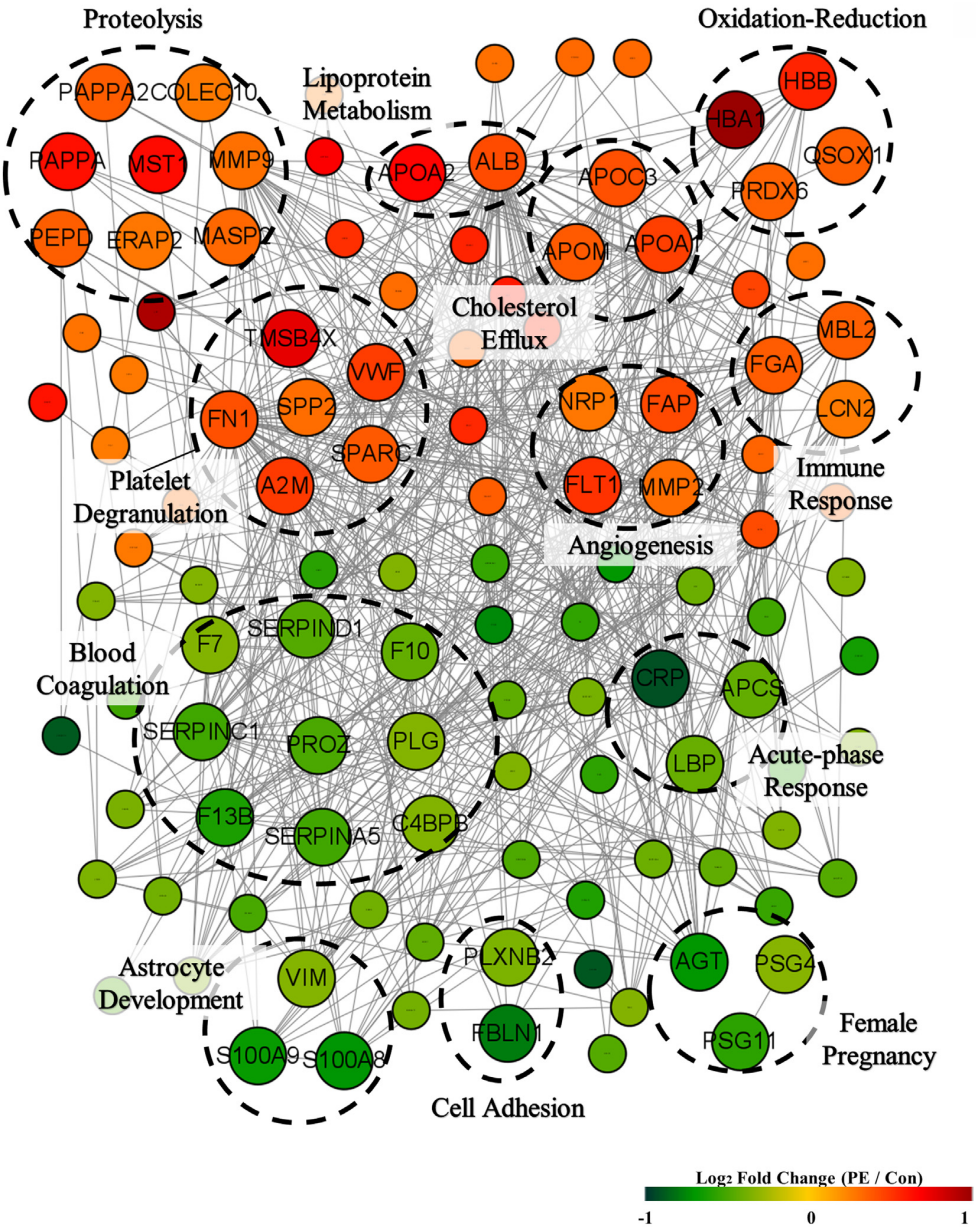
**Interaction networks of differentially expressed proteins in preeclampsia**. Each node represents a protein, and each edge represents an interaction. Red and green represent upregulated and downregulated proteins in the PE group when compared to the control group, respectively. Preeclampsia (PE), Control (Con).

The protein-protein interaction network of the up-and down-regulated DEPs indicated dynamic connections linked to the functional clusters in the biological pathway networks of PE (Fig. 2). The upregulated DEPs in PE indicated the connection of important nodes in biological clusters, which involved proteolysis, lipoprotein metabolism, oxidation-reduction, platelet degranulation, angiogenesis, cholesterol efflux, and innate immune response. The downregulated DEPs in PE were important components in processes that involved blood coagulation, acute-phase response, astrocyte development, cell adhesion, and female pregnancy. This interactive network of DEPs suggests links between biological pathways influencing the various clinical symptoms of PE and allows the selection of novel plasma protein biomarkers for the diagnosis of PE.

### Identification of Plasma Biomarkers for PE

**Fig. 3:**
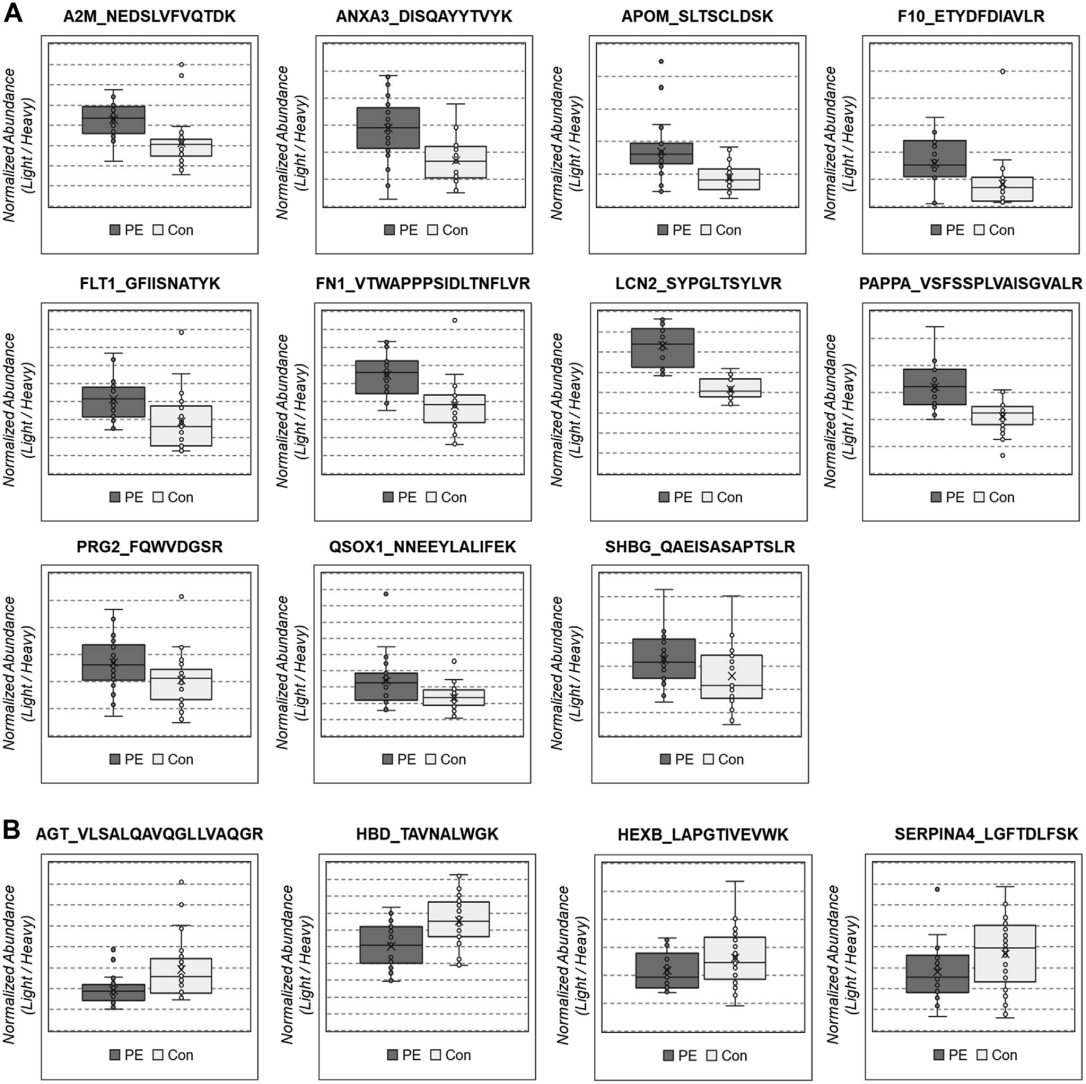
**Analysis of differentially expressed proteins in preeclampsia (PE) by multiple reaction monitoring (MRM).***A*, upregulated proteins in PE. *B*, downregulated proteins in PE. After the protein name, the unique peptide sequence, used in MRM is presented.

**Fig. 4:**
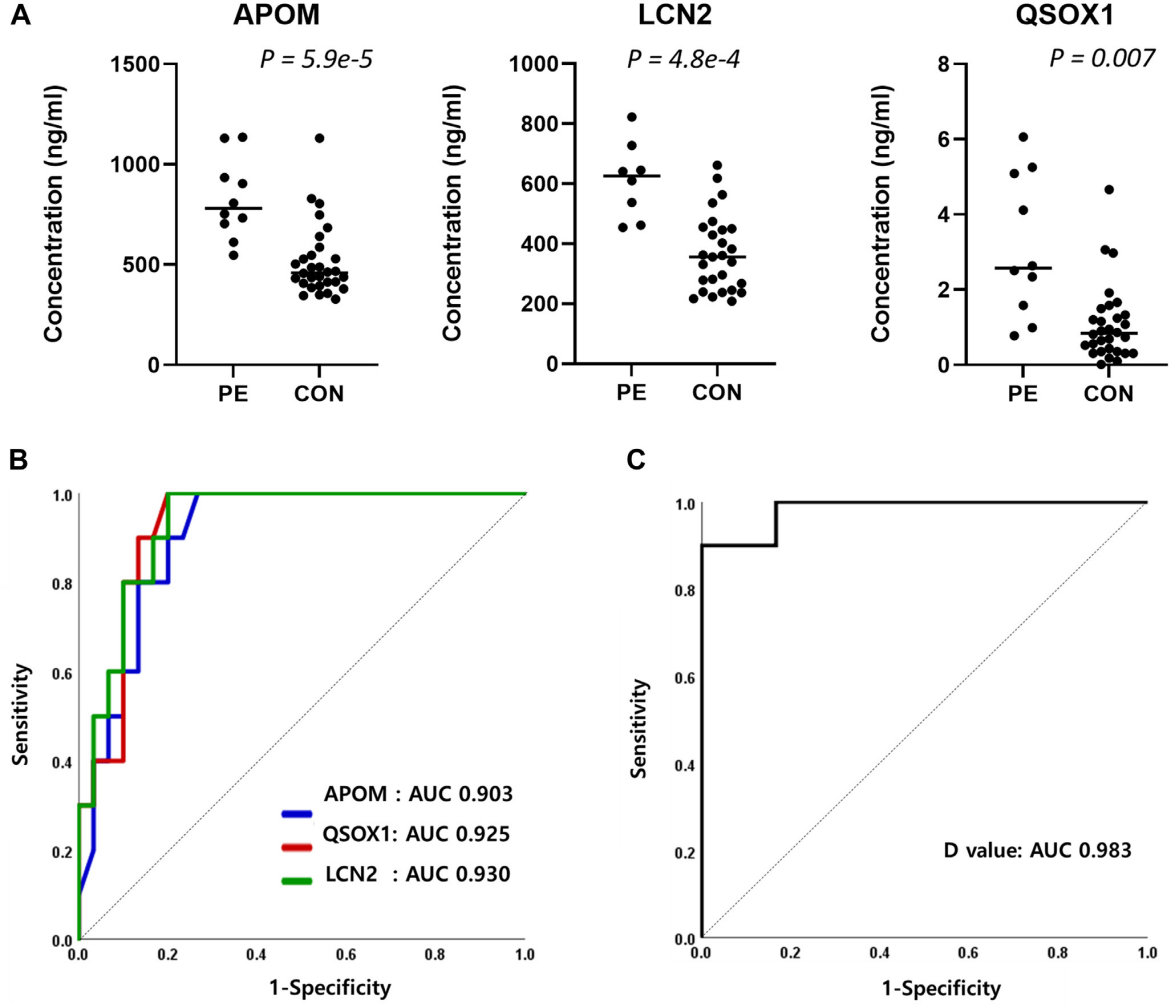
**Significantly increased three proteins in PE, LCN2, APOM, and QSOX1**. *A*, result of ELISA analysis of three markers. *B*, receiver operator characterization (ROC) curve and AUC of the levels of three markers. *C*, AUC of the levels of combination of three markers.

The targeted proteomic assays reported in this manuscript are Tier 2 level, which refers to analysis that use isotope-labeled internal standards for each analyte with nonclinical uses. To identify plasma biomarkers for PE, we selected 41 quantifiable peptides representing 40 proteins among the DEPs using the following criteria: (i) a peptide from proteins identified by at least three peptides, (ii) a unique peptide length of 7–20 amino acids, and (iii) avoid cysteine and methionine (Supplemental Table S4). Expression levels of the candidate proteins were then analyzed using MRM of the selected target peptides in the independent cohort consisting of 29 healthy controls and 26 patients with PE. The levels of the 15 selected candidate proteins were significantly different between the two groups (*p* < 0.05 for all). The levels of A2M, ANXA3, APOM, F10, FLT1, FN1, LCN2, PAPPA, PRG2, QSOX1, and SHBG levels were higher in the PE group than in the control group (Fig. 3*A*), whereas the levels of AGT, HBD, HEXB, and SERPINA4 were lower (Fig. 3*B*). The ROC curves for all biomarker levels were highly significant (*p* < 0.001 for all, Fig. 4).

Among the 11 proteins with increased plasma levels, APOM, LCN2, and QSOX1 were presented as components in the protein-protein interaction network of DEP in PE. In the protein-protein interaction network of DEP shown in Figure 2, APOM was identified as a protein acting on cholesterol efflux, LCN2 as a protein acting on innate immune response, and QSOX1 as a protein acting on oxidation-reduction.

### Clinical Accuracy of Plasma Biomarkers for PE

A case-controlled study was performed to evaluate the diagnostic accuracies of three plasma biomarker candidates: APOM, LCN2, and QSOX1. A total of 40 subjects were included for the validation using ELISA. 10 patients diagnosed with PE and 30 normotensive patients who delivered normal-weight neonates without serious medical or obstetric complications at full term were selected as controls.

The levels of three candidates were significantly increased in the PE cases compared to those in the controls (*p* < 0.001 for all, Table 2 and Fig. 4*A*). These patterns were consistent with those of the proteomic analysis carried out by LC-MS/MS and MRM. There were no significant differences in the levels of the three candidates according to the clinical characteristics such as maternal age, prepregnancy BMI, and nulliparity (*p* > 0.05 for all, Table 3).

**Table 2:** Fold changes of novel biomarkers in PE compared to the Con

Method	Subjects	APOM	QSOX1	LCN2
MRM	Fold Change	1.92	1.43	1.52
	*p* value	<0.001	0.003	<0.001
ELISA	Fold Change	1.60	1.69	2.89
	*p* value	<0.001	0.002	0.001

A comparison of levels was performed using a Student’s *t* test.

**Table 3:** Levels of novel biomarkers related to clinical features

Characteristics	APOM (ng/ml)	QSOX1 (ng/ml)	LCN2 (ng/ml)
Maternal age, y			
<35 (n = 16)	45.6 ± 18.8	1.5 ± 0.4	625.6 ± 228.7
≥35 (n = 24)	40.9 ± 14.1	1.9 ± 0.3	569.4 ± 226.1
*p* value	0.413	0.438	0.450
Prepregnancy body mass index, kg/m^2^			
<25 (n = 34)	40.1 ± 14.5	1.6 ± 0.2	575.6 ± 233.4
≥25 (n = 6)	55.1 ± 20.2	2.4 ± 0.8	684.0 ± 162.5
*p* value	0.145	0.379	0.195
Nulliparous			
Parous (n = 14)	43.0 ± 13.5	1.8 ± 0.4	517.2 ± 144.8
Nulliparous (n = 26)	42.7 ± 17.6	1.7 ± 0.3	632.1 ± 252.8
*p* value	0.959	0.839	0.076

Data are presented as the mean ± standard deviation. A comparison of levels was performed using a Student’s *t* test.

**Fig. 5:**
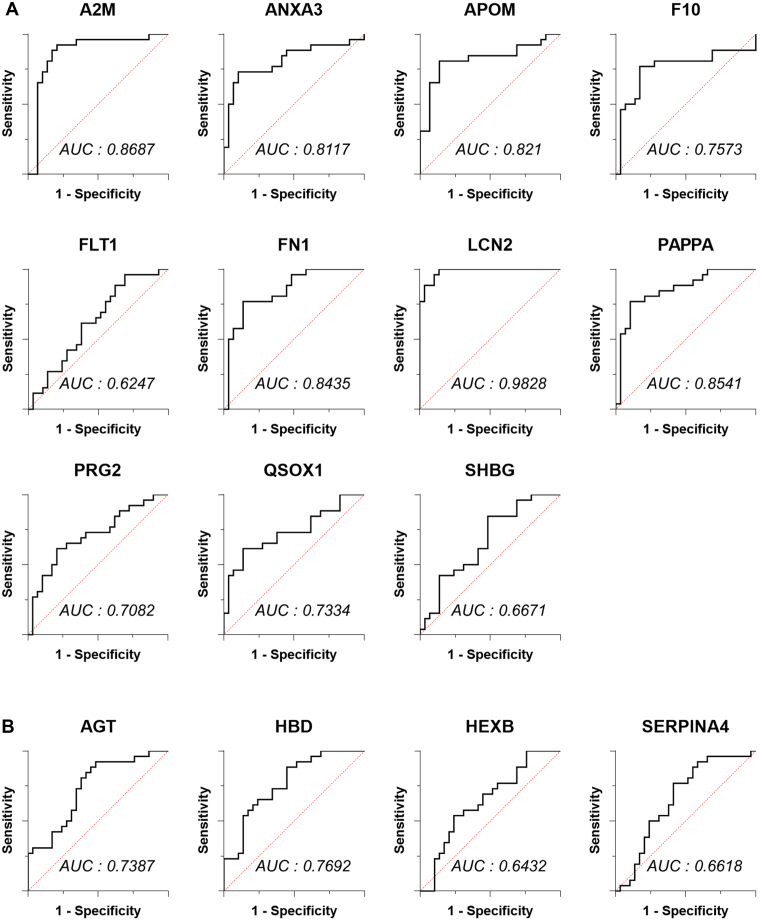
**Receiver Operator Characterization (ROC) curves and AUC values for all differentially expressed proteins.***A*, ROC curves and AUC values for upregulated proteins in PE, *B*, downregulated proteins in PE.

In addition, we analyzed the overall diagnostic accuracy of APOM, LCN2, and QSOX1 levels for PE determination using receiver operator characterization (ROC) curves. The ROC curves are presented in Figure 4*B*. The AUC of the levels of APOM, LCN2, and QSOX1 was 0.903 (95% CI: 0.812-0.995) with a standard error (SE) of 0.047, 0.930 (95% CI: 0.584–1.000) with a SE of 0.039, and 0.925 (95% CI: 0.845–1.000) with a SE of 0.041, respectively. Enhanced performance was achieved by combining the three biomarkers. The AUC was 0.983 (95% CI: 0.947–1.013) for PE *versus* controls (*p* < 0.001, Fig. 4*C*). The cutoff value for each was set at 90% sensitivity by ROC analysis, allowing comparison of the specificity for each biomarker at an equivalent sensitivity (Table 4). The APOM and LCN2 showed a specificity of 83.3%, and QSOX1 showed the highest specificity at 86.7%. The D value by combining the three biomarkers had 100% specificity. The plasma levels of these proteins may add valuable information for the design of multimarker panels for the diagnosis of PE. The ROC curves and AUC values for upregulated proteins in PE are presented in Figure 5*A*, while those for downregulated proteins appear in Figure 5*B*.

**Table 4:** The utility of biomarkers for the detection of PE

The cutoff value	Cutoff	Specificity (%)	PPV (%)	NPV (%)
APOM (ng/ml)	45.9	83.3 (65.3–93.4)	64.3 (44.1–80.4)	96.2 (79.5–99.4)
LCN2 (ng/ml)	599.74	83.3 (65.3–93.4)	64.3 (44.1–80.4)	96.2 (79.5–99.4)
QSOX1 (ng/ml)	1.72	86.7 (69.3–96.2)	69.2 (46.9–85.2)	96.3 (80.1–99.4)
D value	−8.09	100.0 (88.4–100.0)	100.0 (66.4–100.0)	96.8 (82.4–99.5)

The cutoff was set at 90% sensitivity by ROC curve analysis. To compare detection accuracies of factors, specificity, positive predictive value (PPV), and negative predictive value (NPV) were calculated at equivalent sensitivity. The number in parentheses indicates the 95% confidence interval.

## Discussion

Recent achievements in proteomics have opened a new chapter in protein biomarker discovery, contributing to diagnosis in clinical practice. However, novel marker proteins are rarely used clinically due to their limited reproducibility and specificity (16). In this study, we used an analytic technique incorporating LC-MS/MS and MRM for the development of plasma biomarker proteins in pregnant women with PE. The various DEPs in plasma samples from a cohort with PE were identified by LC-MS/MS-based large-scale and MRM-based targeted quantitative proteomic analyses. Moreover, the expression patterns of the LC-MS/MS and MRM-based DEPs were in agreement with those observed using ELISA. Therefore, these DEPs can be considered effective plasma biomarkers for PE diagnosis, regardless of the clinical characteristics of the pregnant women, such as maternal age, prepregnancy BMI, and parity. These results suggest that the LC-MS/MS and MRM-based quantitative proteomic analyses may be useful for the identification of plasma biomarkers in various diseases.

In this study, we identified several plasma proteins as potential diagnostic biomarkers for PE using proteomics technologies. Among them, APOM, LCN2, and QSOX1, related to the biological function clusters in the pathway networks of PE, were found to possess high diagnostic accuracy. Furthermore, the combination of these three biomarkers increased the diagnostic accuracy for PE detection to AUC 0.98.

Previous studies have reported that APOM plays an essential role in defective deep placentation by controlling inflammatory responses and lipid transport (17). Regarding the risk of PE, several recent studies have reported changes in cholesterol homeostasis and metabolism. HDL cholesterol efflux capacity, as the ability of HDL to remove cholesterol from macrophages, has been reported as a new biomarker for cardiovascular risk, and also for PE (18, 19). It has been suggested that endothelial dysfunction in PE could be attributed to the accelerated lipid metabolism outbalancing the remnant removal mechanisms. However, the relationship between altered cholesterol homeostasis/metabolism and PE should be evaluated in further studies. Our results may provide additional insight into the pathophysiology of PE generated by changes in cholesterol homeostasis and metabolism mediated by APOM.

LCN2 is a secretory glycoprotein implicated in many functions such as apoptosis and innate immunity. It has been recognized to have potential effects on obesity, inflammation, and insulin resistance. However, many controversial studies about the changes in plasma LCN2 levels in PE have been reported (20, 21). A study using a PE animal model showed that increased LCN2 plasma levels can be used as an indicator of renal complications and coagulopathies in PE (22). In that study, the authors suggested that PE patients presenting highly elevated LCN2 levels should be continuously assessed for renal functions and hemostatic parameters. In other studies, it has been reported that circulating LCN2 levels were upregulated because of the generalized endothelial injury associated with PE (23, 24). In agreement with these findings, our results showed that plasma LCN2 levels in the PE group were significantly higher than those in the control group. These results further support the evidence that LCN2 expression is increased in PE.

QSOX1 is a member of the QSOX family that belongs to a class of flavin adenine dinucleotide (FAD)-dependent thiol oxidases. It promotes the formation of disulfide bonds in peptides and proteins and reduces molecular oxygen (O_2_) to hydrogen peroxide (H_2_O_2_) (25). H_2_O_2_ is a known oxidative stress inducer that promotes apoptosis in primary cultured trophoblasts (26). In previous studies, placentae from pregnancies complicated by PE have been associated with hypoxia and elevated oxidative stress (27, 28, 29, 30). Moreover, QSOX1 is highly expressed in term placenta of both normal and PE pregnancies, implying that it may play a critical role in placental development (31). These results suggest that hypoxia-induced upregulation of QSOX1 and a consequent elevation in intracellular H_2_O_2_ levels may lead to the increment of apoptosis in the placentae of pregnancies complicated by PE. However, changes in QSOX1 expression in the plasma of pregnant women with PE are yet to be reported. Our results demonstrated that QSOX1 levels were upregulated in the maternal plasma of the PE group compared to those of the control group. To the best of our knowledge, this study is the first study to analyze changes in QSOX1 expression in the maternal plasma of pregnant women with PE. Our findings warrant further investigation to address the association of QSOX1 with the pathogenesis of PE.

In conclusion, the DEPs identified by systematic proteome profiling were shown to be associated with various biological processes of PE. Among them, APOM, LCN2, and QSOX1 exhibited significant diagnostic accuracy for PE using plasma samples of pregnant women. These proteins may play a key role in the pathophysiology of the disease and could aid in the effective diagnosis of PE. Further studies may support the functional significance of our proteomic investigation in the pathophysiology of PE.

### Perspectives

PE is one of the leading causes of maternal morbidity and mortality all over the world. The clinical phenotype varies, with symptoms ranging from increased blood pressure to more serious complications, including renal and liver dysfunction and seizures. However, the causes and pathophysiology of PE largely remain undefined. In the past decade, various biomarkers have been developed and studied in an attempt to aid in the diagnosis of PE and to improve upon the clinical signs and symptoms. Here, we performed systematic proteome profiling of the plasma of pregnant women with and without PE using LC-MS/MS and MRM analysis and found three effective plasma biomarkers with high diagnostic accuracy for PE. Our results suggest that the LC-MS/MS and MRM-based quantitative proteomic analyses may be useful for the identification of plasma biomarkers in various hypertensive disorders.

## Data Availability

The mass spectrometry proteomics data have been deposited to the ProteomeXchange Consortium *via* the PRIDE partner repository with the dataset identifier PXD050118 and 10.6019/PXD050118.

The targeted mass spectrometry proteomics data have been deposited to the PASSEL consortium *via* the PeptideAtlas partner repository with the dataset identifier PASS05863.

## Supplemental data

This article contains supplemental data.

## Conflict of interests

The authors declare that they have no conflicts of interest with the contents of this article.
